# Sustainable PET Waste Recycling: Labels from PET Water Bottles Used as a Catalyst for the Chemical Recycling of the Same Bottles

**DOI:** 10.1021/acssuschemeng.3c04997

**Published:** 2023-11-06

**Authors:** Mojtaba Enayati, Somayeh Mohammadi, Martin G. Bouldo

**Affiliations:** Center for Materials and Manufacturing Sciences, Departments of Chemistry and Physics, Troy University, Troy, Alabama 36082, United States

**Keywords:** waste to value, packaging labels, thermolysis, chemical recycling, poly(ethylene terephthalate), catalytic depolymerization

## Abstract

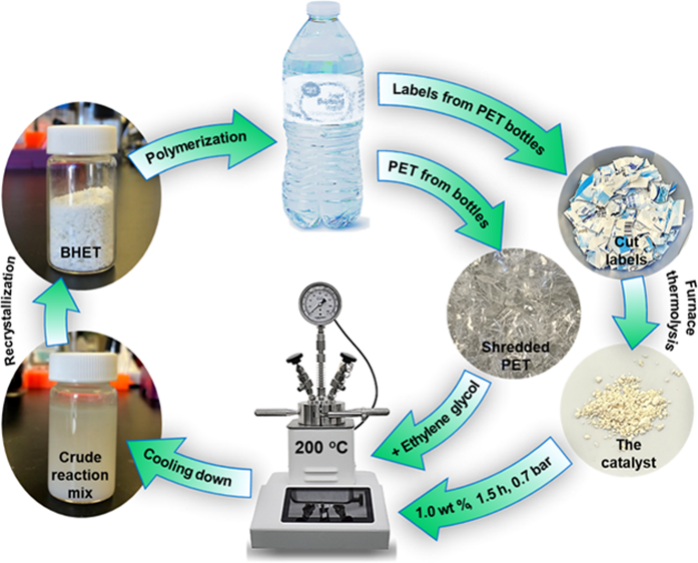

We report using a waste material, poly(ethylene terephthalate) (PET) water bottle labels, for the chemical recycling of the same PET water bottles. The solid fillers used for the manufacturing of the packaging labels were recovered by thermolysis in an electrical furnace at 600, 800, and 1000 °C with 13.5, 12.0, and 10.4 wt % recovery. Characterization of the solid residue showed the presence of calcium carbonate, calcium oxide, and titanium dioxide, which are typical fillers used for packaging film manufacturing, such as water bottle labels. These solid residues were then used as a catalyst for PET depolymerization by glycolysis, in which the catalyst recovered from bottle labels and shredded PET reacted in the presence of excess ethylene glycol at 200 °C. The reaction mixtures were analyzed for PET conversion and the yield of the bis(2-hydroxyethyl)terephthalate (BHET) monomer as the final product of the glycolysis reaction to determine the efficiency of the catalyst. Our results show that the catalyst prepared at 800 °C (Cat-800) has the best performance and provides a 100% PET conversion with a 95.8% BHET yield with a 1.0 wt % loading in 1.5 h. The catalyst from the PET water bottle labels is nontoxic, readily available, cost-effective, environmentally friendly, and can be used as a model for the self-sufficient chemical recycling of PET via glycolysis.

## Abstract 2

This research demonstrates how everyday items, such as bottle labels, have the existing necessary components for sustainable PET waste recycling.

## Introduction

Plastic pollution has quickly turned into a critical environmental problem that needs to be addressed by governments, industries, and society. Plastics and microplastic contaminants are already present in the soil, drinking water, and aquatic ecosystems and are rapidly accumulating in living organisms,^1^ which can eventually lead to high levels of microplastics in every level of the food chain, threatening human health.^2^ Poly(ethylene terephthalate) (PET) is the third most used plastic in the packaging industry and one of the most used commodity plastics in food and beverage packaging.^3^ A majority of food products such as eggs, fruits, vegetables, bakery products, beverages, and drinking water are currently packaged in PET containers, so much so that 44.7% of the single-used beverage containers in the US and 12% of the global solid waste is PET packaging.^4^ As a result, PET microplastics are increasingly being recognized as a threatening contaminant, resulting in many attempts to substitute this nonbiodegradable polyester with biodegradable alternatives.^5−8^ However, PET is still highly demanded by both industry and consumers due to its low price, excellent mechanical properties, transparency, and performance. Therefore, finding novel and efficient methods for the recycling and upcycling of PET is of great importance.

Mechanical and chemical recycling are the two major methods that are currently available for alleviating some of the environmental burden of plastics such as PET. While mechanical recycling is widely used and is more favored both economically and technologically, it poses significant challenges because of the low thermal stability of PET. At its processing temperature (ca. 270 °C), thermal degradation will result in a drop in molecular weight and mechanical properties of the recycled PET.^9^ Alternatively, although chemical recycling is not cost-effective at a low scale, it leads to various starting materials that can be used for the production of regenerated virgin PET with no trade-off in its mechanical properties or to produce other value-added products.^10^ Several chemical recycling methods for PET are available, which differ in the chemicals used to produce a variety of products from the starting PET waste. Basic, acidic, and enzymatic hydrolysis, alcoholysis with different alcohols such as methanol and 2-ethylhexanol, glycolysis mostly with ethylene glycol (EG), and aminolysis with different amines such as methyl amine and ethanol amine are the major studied routes for PET chemical recycling.^11^ However, these processes need a proper catalyst and design and development of more effective catalysts for the chemical recycling of PET is an active area of interest for scientists, and new catalysts that can perform PET chemical depolymerization easier, more efficiently, and less energy-intensively are introduced frequently.^12−15^ Numerous homogeneous and heterogeneous catalysts have already been developed and used for PET chemical depolymerization, including common acids and bases, solid acids, metal oxides, metallic salts, organometallics, ionic liquids, and enzymes.^13^ For the PET glycolysis depolymerization using EG, which produces bis(2-hydroxyethyl)terephthalate (BHET) monomer, metal acetates including Zn, Mn, Co, and Pb acetates are among the oldest and most used catalysts,^16^ while metal oxides, ionic liquids, and their combinations were studied more recently.^13^ PET glycolysis reactions are usually performed at the boiling temperature of EG, 196 °C; however, other temperatures ranging from 170 to 300 °C have been studied too.^17,18^ Choosing a proper catalyst is critical for reaching complete PET conversion and the highest BHET yield in the lowest amount of time, temperature, and pressure possible.

There has been growing interest, in recent years, in exploring the potential of low-cost, environmentally friendly, and waste-driven catalysts for PET glycolysis. Notably, iron(III) oxide,^19^ doped titanium dioxide,^20^ sodium acetate,^21^ sodium carbonate,^22,23^ calcium carbonate,^21^ commercial calcium oxide,^24^ and even calcium oxide from chicken eggshell, ostrich eggshell, oyster shell,^25,26^ and orange peel ash^27^ have emerged as promising candidates for PET recycling, as they offer a nonhazardous and low-cost alternative to complex synthetic catalysts. The findings from these reports suggest the feasibility of these catalysts in the depolymerization of PET waste into valuable materials, including the BHET monomer.

Considering the catalytic activity of the calcium carbonate (CaCO_3_), calcium oxide (CaO), and titanium dioxide (TiO_2_) and the fact that CaCO_3_ and TiO_2_ are used as solid fillers in labels’ manufacturing for food packaging^28,29^ inspired us to examine the labels from PET water bottles to assess their efficacy as a catalyst for PET depolymerization via glycolysis. In this work, we used the solid residue from high-temperature thermolysis of the labels from PET water bottles in an electrical furnace as a catalyst for the glycolysis of the same PET bottle waste in order to produce the BHET monomer. Our results showed that by using 1.0 wt % of this catalyst, a PET conversion of 100% and a BHET yield of 95.8% can be reached in 1.5 h at 200 °C. We observed a strong relationship between the thermolysis temperature and the catalyst efficiency in the PET glycolysis reaction.

## Materials and Methods

### Materials

Clean commercial 500 mL PET water bottles from a single brand were acquired from a local market for the experiment. The bottles had their caps removed, and their labels were set aside for catalyst preparation. The bottles were then sufficiently dried before being cut into 2–5 mm flakes via an industrial shredder (Brabender CWB, Granu-Grinder M120/150). The shredded flakes were triple-washed with methanol and dried at 60 °C to be used throughout the study. Ethylene glycol (EG) and methanol were purchased from Fisher Chemical. CaCO_3_ (98%) from Thermo Scientific and CaO (99%) and TiO_2_ (99.5%) from Sigma-Aldrich were used as catalysts for control experiments. BHET was acquired from Sigma-Aldrich and used as a standard for product characterization and HPLC calibration. All chemical reagents were designated as analytically pure and used without additional purification.

### Characterization

Characterization of solid residues from incinerated labels as catalysts and the PET glycolysis products was conducted using Fourier transform infrared (FTIR) spectroscopy, nuclear magnetic resonance (NMR), thermogravimetric analysis (TGA), differential scanning calorimetry (DSC), high-pressure liquid chromatography (HPLC), X-ray diffraction (XRD), scanning electron microscopy (SEM), and energy dispersive X-ray spectroscopy (EDX). A PerkinElmer, Waltham, MA, FTIR spectrophotometer was used to determine the main functional groups in the wavenumber range of 400–4000 cm^–1^. The resolution was set at 4 cm^–1^, and 64 scans were conducted. ^13^C NMR and ^1^H NMR spectral data investigating the glycolyzed product structure were obtained via an Ascend TM (Bruker, Switzerland) spectrometer (400 MHz). Tzero Pans were used with a DSC-250 (TA Instruments, DE) in order to measure thermal transitions with a heating rate of 10 °C/min from 40 to 300 °C. Analysis of thermal stability was conducted by a TGA-550 (TA Instruments, DE) utilizing platinum-HT sample pans. A heating rate of 20 °C/min from room temperature to 800 °C under nitrogen was used for TGA studies. High-performance liquid chromatography (HPLC, Shimadzu, LC-10AT, UV–vis detector) was used to determine the qualitative composition of products as well as the quantitative BHET yield. The HPLC utilized a Poroshell 120 column (EC-C18, 2.7 μm, 3.0 mm × 150 mm) and a mobile phase of methanol (75%) and H_2_O (25%), with a column temperature of 25 °C, a flow rate of 0.2 mL/min, and an injection volume of 5 μL. The UV detector was set to 254 nm. The SEM-EDX analysis was conducted using a Carl Zeiss SMT, Inc. instrument with an applied voltage of 20 kV. An X-ray diffraction (XRD) pattern was obtained using a Bruker D-8 powder diffractometer. The XRD analysis was performed at 40 kV with a copper radiation source, a slit width of 0.6 mm, a scan speed of 0.1 s/step, and a step increment of 0.01°. Nitrogen adsorption/desorption analysis was performed by a Micrometrics ASAP 2020 volumetric adsorption analyzer at 77 K to measure the specific surface area of the catalysts via the Brunauer, Emmett, and Teller (BET) method. Samples were degassed for 24 h at 90 °C before BET measurement.

### Catalyst Preparation from Water Bottle Labels

**Figure 1:**
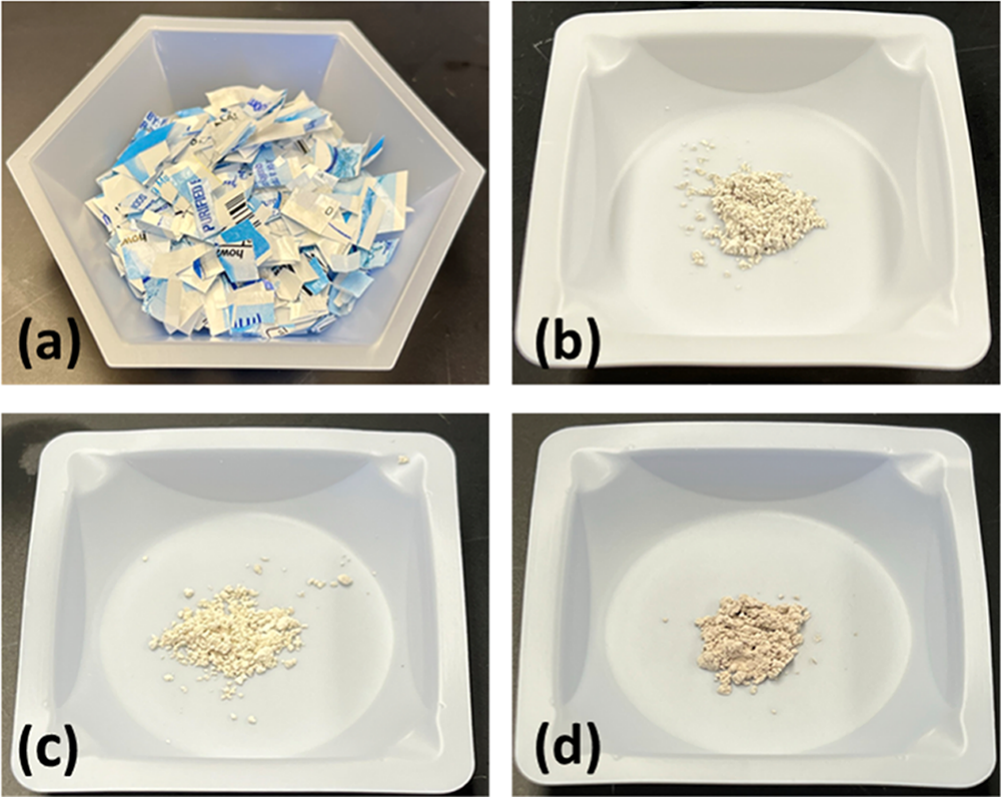
Cut labels from PET water bottles before thermolysis (a) and the residual powder (catalysts) after thermolysis of the labels in the furnace at 600 °C (Cat-600) (b), 800 °C (Cat-800) (c), and 1000 °C (cat-1000) (d).

Labels were detached from each PET bottle and then cut into strips sized about 2 cm × 0.5 cm (Figure 1a). The cut labels were packed into a small crucible, weighed, and then added to an electrical muffle furnace (Yamato, FO200CR) at room temperature. The furnace was set to the desired temperature of 600, 800, or 1000 °C to be held for 2 h in order to ensure complete thermal decomposition of the label material. Once the heat treatment phase was concluded, the furnace was allowed to cool to room temperature. Afterward, the crucibles were removed from the furnace and the residual powder was collected and weighed (Figure 1b–1d). A ratio was then computed from the original mass of the labels and residual powder after thermolysis. For the thermolysis at 600 °C (Cat-600), we found 13.5 wt % of the residual powder based on the initial weight of the labels, while for 800 (Cat-800) and 1000 °C (Cat-1000), the solid weight recovery was 12.0 and 10.4 wt %, respectively.

### PET Depolymerization via Glycolysis

**Figure 2:**
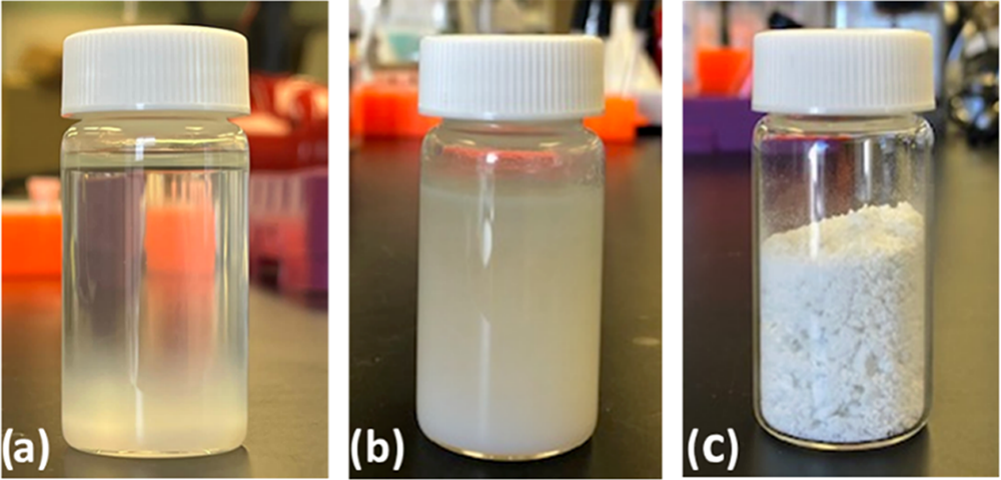
Reaction mixture of PET glycolysis at 70 °C containing the dispersed catalyst Cat-800 (a), the reaction mixture after cooling to room temperature (b), and BHET crystals from recrystallization in cold water (c).

**Scheme 1:**
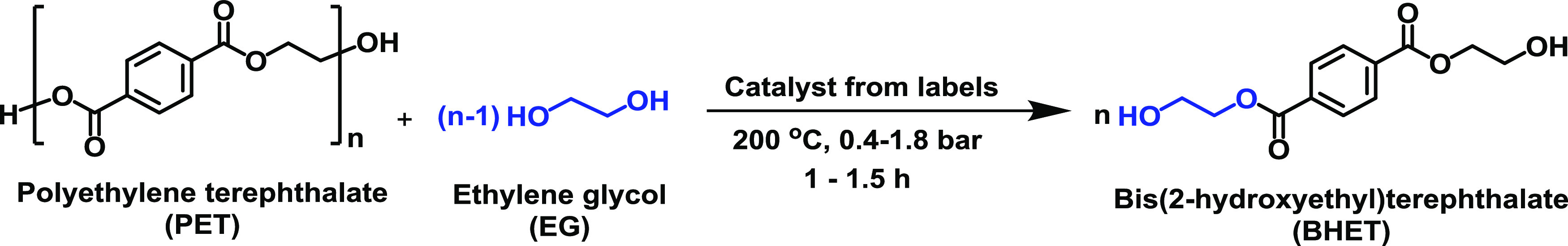
PET Glycolysis Using a Catalyst by the Thermolysis of the Labels from PET Bottles

A high-pressure hydrothermal autoclave reactor (100 mL) equipped with a Teflon vessel, a pressure and temperature monitor, and a magnetic stirrer was used to conduct glycolysis reactions. In a typical glycolysis reaction, the required amounts of PET, EG, and Cat-600, Cat-800, or Cat-1000 were weighed and added to the Teflon vessel, which was then inserted into the reactor. The reactor was closed and heated from room temperature to 200 °C, taking about 2 h, and was kept at 200 °C for the required amount of time (Scheme 1). The heating was then turned off, and the reaction was cooled to around 75 °C, at which point the reactor was opened and the reaction mixture was visually inspected. In the cases with high BHET yields, the reaction mixture was a one-phase liquid solution at 70–75 °C with the white catalyst dispersed in it (Figure 2a). The dispersed catalyst was removed from the hot solution by vacuum filtration. However, preheating the funnel is necessary to prevent BHET and oligomers from precipitating onto the inner walls of the filtration apparatus because their solubility in EG is temperature-dependent. Upon cooling the filtered reaction mixture to room temperature, the BHET and the other possible products, i.e., PET oligomers, were precipitated as a white solid (Figure 2b). A small portion of this two-phase mixture was vacuum-filtered to remove the excess EG and vacuum-dried at 65 °C overnight to be used as a crude reaction mixture for reaction characterization and BHET yield measurements via HPLC. The reaction mixture was poured into cold water and kept at 4 °C overnight for the BHET to be crystallized, which then was filtered and vacuum-dried at 65 °C (Figure 2c). When the PET conversion is incomplete, in which there are remaining deformed flakes of PET, the hot reaction mixture is filtered through a hot funnel to separate and dry the unreacted PET. The weight of this unreacted PET was then measured, and the PET conversion was calculated using the following equation (eq 1)
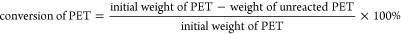
1

## Results and Discussion

### Characterization of the Catalysts

**Figure 3:**
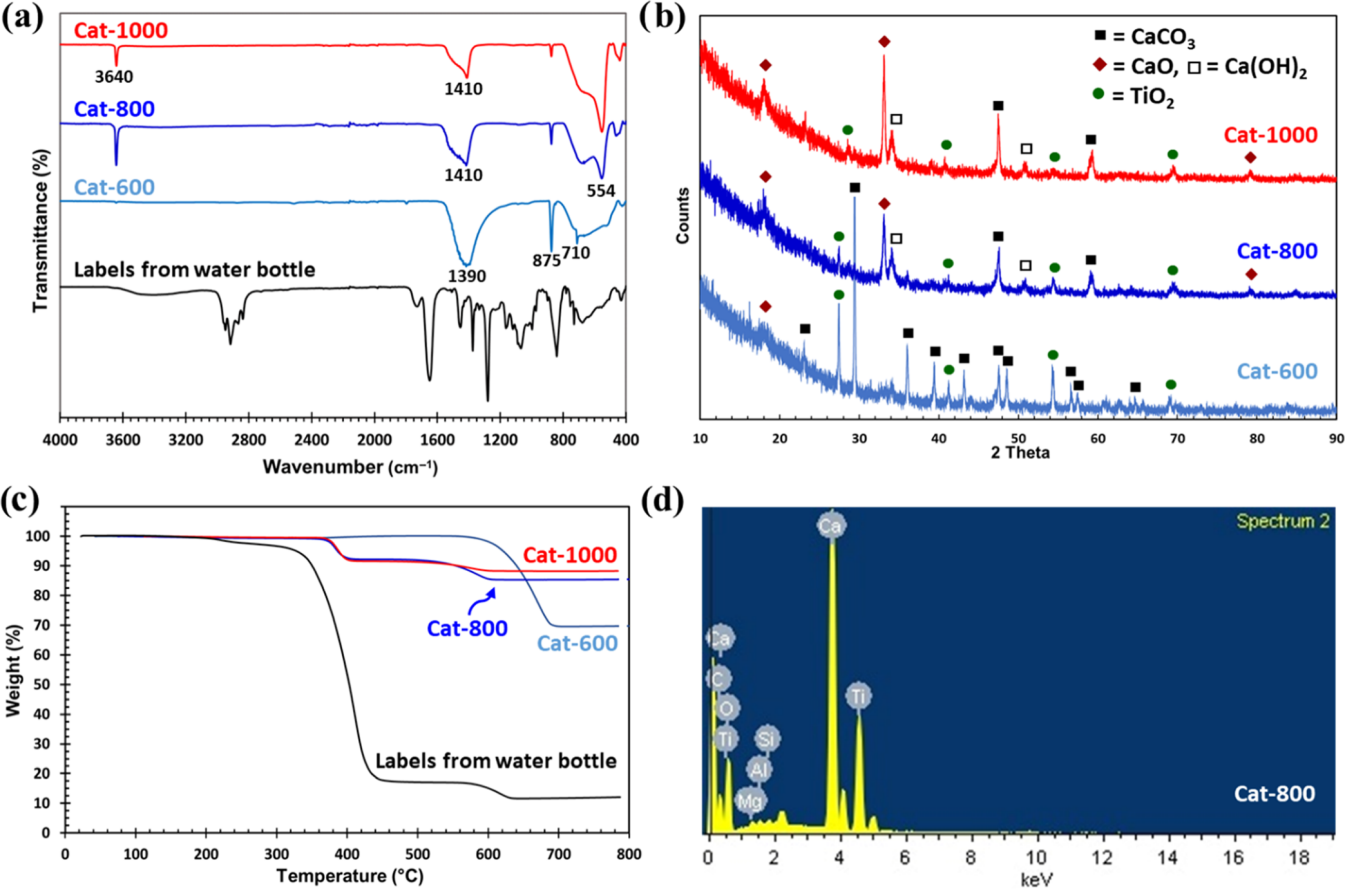
FTIR spectra of the label and three catalysts (a), XRD patterns for the three catalysts (b), TGA thermograms of the label and three catalysts (c), and the EDX spectrum of Cat-800 (d).

We used Fourier transform infrared spectroscopy (FTIR), thermogravimetric analysis (TGA), X-ray diffraction (XRD), scanning electron microscopy (SEM), and energy dispersive X-ray spectroscopy (EDX) to characterize the catalyst prepared from waste labels. Figure 3 shows the FTIR spectra, XRD patterns, TGA thermograms, and EDX of the labels from PET water bottles and the three catalysts that were prepared at 600, 800, and 1000 °C. The FTIR of the labels (Figure 3a) shows characteristic peaks of the PET/polypropylene film that is usually used in the fabrication of labels for food packaging.^30^ The FTIR of the three catalysts (Figure 3a) shows peaks at 710, 875, and 1390 cm^–1^ for the calcium carbonate (CaCO_3_), which shifted to around 1440 cm^–1^ for samples with higher-temperature thermolysis, indicating the change of the calcite polymorph to aragonite and the partial conversion of CaCO_3_ to calcium oxide (CaO)^31,32^ and a broad peak at 440–820 cm^–1^ for titanium dioxide (TiO_2_).^33^ The sharp peak at 3640 cm^–1^ that was observed for 800 and 1000 °C samples is attributed to Ca(OH)_2_, which is a result of the adsorption of water by CaO and/or surface OH groups.^34,35^ XRD patterns of the catalyst prepared at 600 °C (Figure 3b) clearly show CaCO_3_ peaks as well as some of the CaO and heat-treated titanium dioxide (TiO_2_).^33,36,37^ As the temperature of the thermolysis in the furnace increased to 800 and 1000 °C, the peaks associated with CaCO_3_ are diminished and the sample shows more characteristic peaks of CaO, its hydrated product Ca(OH)_2_,^38^ as well as the peaks for heat-treated TiO_2_.^33,37^ The increasing CaO content with the temperature increase is not unexpected because it is well-known that CaCO_3_ starts losing carbon dioxide (CO_2_) at higher temperatures and converts to CaO.^32,39^ This is further confirmed by increasing weight loss with temperature during the preparation of the catalyst by thermolysis, as we recovered 13.5, 12.0, and 10.4 wt % of the solid content from labels at 600, 800, and 1000 °C, respectively. TGA thermograms of the label show the thermal behavior of PET/polypropylene multilayer films^40^ with the highest weight loss at 412 °C (Figure 3c). The catalyst prepared at 600 °C shows a 30.25 wt % weight loss at 675 °C that is due to CO_2_ loss upon heating. The two catalysts prepared at 800 and 1000 °C both showed almost similar thermal behavior with a total 14.54 and 11.73 wt % weight loss, respectively, at two temperature steps of 390 (loss of water from Ca(OH)_2_)^34^ and 580 °C (loss of CO_2_ from CaCO_3_) (Figure 3c). Figure 3d shows the EDX spectrum of the catalyst prepared at 800 °C, which indicates peaks associated with oxygen, calcium, titanium, and carbon. These results are consistent with the semiquantitative average weight percentages of the element in catalysts prepared at different temperatures by EDX, which is shown in Table 1. As shown in Table 1, carbon (C), oxygen (O), calcium (Ca), and titanium (Ti) make up 99.2 wt % of the three catalysts, which again confirms the presence of CaCO_3_, CaO, and TiO_2_. While the total average of these four elements is the same for all three catalysts, the individual percentages changed dramatically for Cat-600 compared to Cat-800 and Cat-1000 as the weight percentage of carbon decreased and calcium and titanium increased for Cat-800 and Cat-1000 compared to Cat-600. This is another indication of the removal of CO_2_ from CaCO_3_ at higher temperatures that reduces the carbon weight percent in the catalyst. As shown in Figure 3 and Table 1, the structural changes in the composition of the catalyst by increasing the thermolysis temperature from 800 to 1000 °C are not significant and therefore have not been studied in more detail.

**Table 1:** Semiquantitative Average Weight Percentage of the Element in Catalysts Prepared at Different Temperatures

	Cat-600	Cat-800	Cat-1000
element	average weight %	average weight %	average weight %
C	15.99	8.25	7.69
O	47.19	46.37	47.01
Mg	0.41	0.33	0.31
Al	0.22	0.25	0.23
Si	0.19	0.22	0.23
Ca	24.00	28.84	30.32
Ti	12.02	15.75	14.21

**Figure 4:**
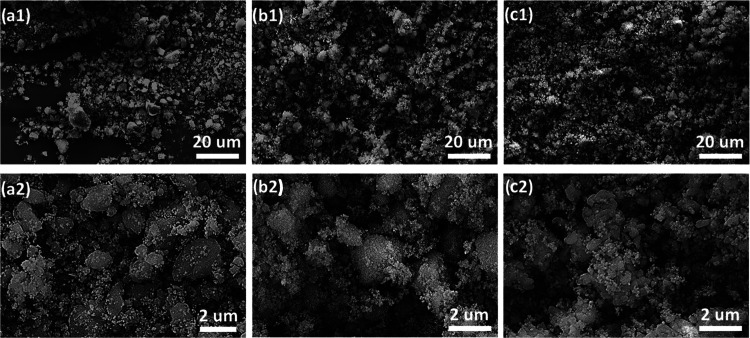
SEM micrographs of Cat-600 (a1, a2), Cat-800 (b1, b2), and Cat-1000 (c1, c2) in two different magnifications.

The morphology and particle size of the three catalysts were studied using SEM. Figure 4 shows the SEM micrographs of the catalysts prepared from the labels at two different magnifications. All samples show a bimodal distribution of particle sizes where there are bigger particles of 1–5 μm mixed with smaller submicron particles. However, with the increasing temperature of the thermolysis, the particle size decreased, as shown by the SEM micrographs of Cat-800 and Cat-1000 (Figure 4b1,b2,c1,c2).

The specific surface areas of the three catalysts were also measured using nitrogen adsorption/desorption to study the possible correlation of their surface areas with their catalytic performance. The specific surface areas for Cat-600, Cat-800, and Cat-1000 are 0.3868, 4.1296, and 0.4989 m^2^/g, respectively, which shows that these catalysts from label thermolysis are nonporous. However, the Cat-800 catalyst showed a higher surface area as compared to those of Cat-600 and Cat-1000, probably due to a smaller particle size (Figure 4b).

### PET Glycolysis Reactions Using the Catalyst from Label Thermolysis

#### Catalysts’ Efficiency for the PET Glycolysis Reaction

The prepared catalysts were used in PET glycolysis reactions to study their effectiveness and catalytic activity in PET depolymerization with excess EG. Table 2 shows the results of PET glycolysis reactions using Cat-600, Cat-800, and Cat-1000 compared to the results from the control experiment using commercially available CaCO_3_, CaO, and TiO_2_.

**Table 2:** Reaction Conditions, PET Conversion, and BHET Yield for PET Glycolysis Using Catalysts from Label Thermolysis^a^

run	catalyst and loading (wt %)	time (h)^a^	pressure (bar)^b^	PET conversion (%)	BHET yield (%)
blank	No catalyst	1.0	0.4	27	10.5 ± 0.10
L1	Cat-600 (1.0%)	1.0	0.4	71	25.1 ± 0.37
L2	Cat-600 (5.0%)	1.0	1.8	97	70.3 ± 0.43
L3	Cat-800 (5.0%)	1.0	1.2	100	79.5 ± 0.55
L4	Cat-800 (3.0%)	1.0	0.7	100	82.2 ± 0.44
L5	Cat-800 (1.0%)	1.0	0.4	100	81.9 ± 0.27
L6	Cat-800 (1.0%)	1.5	0.7	100	95.8 ± 0.11
L7	Cat-800 (0.5%)	1.5	0.3	95	80.1 ± 0.35
L8	Cat-1000 (1.0%)	1.0	0.8	93	64.6 ± 0.06
L9	Cat-1000 (2.0%)	1.0	0.6	100	81.9 ± 0.45
control 1	CaCO_3_ (1.0%)	1.5	0.6	82	57.3 ± 0.53
control 2	CaO (1.0%)	1.5	0.3	100	95.0 ± 0.32
control 3	TiO_2_ (1.0%)	1.5	1.9	86	43.5 ± 0.45

a All reactions were performed with an EG:PET ratio of 5.0 (w/w) at 200 °C.

b This is the highest pressure of the reaction during the glycolysis.

As the results in Table 2 show, Cat-600 cannot provide a complete PET conversion at a 1.0 or 5.0 wt % loading where it shows only a 71 and 97% PET conversion and a 25.1 and 70.3% BHET yield for 1.0 and 5.0 wt %, respectively (Table 2, entries L1 and L2). Cat-800, however, showed much better efficiency for PET conversion, as we found that this catalyst can provide a 100% PET conversions at 5.0, 3.0, and 1.0 wt % loadings with almost the same amount of the BHET yield of around 80% for 1 h at 200 °C (Table 2, entries L3–L5). It is a significant result that Cat-800, which is prepared from the waste labels, can depolymerize PET via glycolysis with complete conversion and a high yield of BHET of 81.9% at a loading of 1.0 wt % in 1 h. In an attempt to increase the BHET yield using this catalyst, we increased the time of the reaction from 1 to 1.5 h (Table 2, entry L6), which showed significant improvement in the BHET yield from 81.9% for 1 h reaction time to 95.8% for 1.5 h. By lowering the Cat-800 loading from 1.0 to 0.5 wt % with the same 1.5 h reaction time, the PET conversion of 95% and the BHET yield of 80.1 were achieved (Table 2, entry L7), suggesting that the optimum amount of Cat-800 loading is 1.0 wt % for the PET glycolysis. Cat-1000 was also used for PET glycolysis, which shows a 93% PET conversion and a 64.6% BHET yield for a 1.0 wt % catalyst loading and a 100% PET conversion and an 81.9% BHET yield for a 2.0 wt % catalyst loading (Table 2, entries L8 and L9).

To compare our best result from Cat-800 in the L6 experiment with the pure alternatives, we used commercially available and pure CaCO_3_, CaO, and TiO_2_ for the PET glycolysis reactions as control experiments with a 1.0 wt % loading in 1.5 h (Table 2). As can be seen from these control experiments, pure CaCO_3_ and TiO_2_ alone cannot provide a complete PET conversion in the same reaction conditions as Cat-800, and they show 57.3 and 43.5% BHET yields, respectively (Table 2, control 1 and control 3). CaO, however, shows a 100% PET conversion and a 95.0% BHET yield with a 1.0 wt % loading in 1.5 h, demonstrating its higher catalytic efficiency for PET glycolysis compared with CaCO_3_ and TiO_2_. This is consistent with the fact that Cat-800 shows better results compared to Cat-600 as it contains higher CaO content.

The performance of a catalyst depends on various factors, including the surface area, crystalline structure, and active sites. The particular arrangement of atoms in the crystal lattice, a higher surface area that offers more active sites for reactant molecules to interact, and an optimal porosity allowing for enhanced diffusion of reactants and products can all influence the catalytic performance of a catalyst. The higher relative specific surface area and the higher amounts of CaO in Cat-800 are the major factors that enhance its catalytic activity compared to Cat-600. As it was discussed before, the structural differences of the catalysts are not significant when the thermolysis temperature increased from 800 to 1000 °C. However, the 1000 °C treatment might have a detrimental effect on the polymorphs of CaCO_3_, CaO, and TiO_2_ in the catalyst, which thereby decreases its efficiency for PET glycolysis. The specific surface area for Cat-1000 is also lower compared to Cat-800 (0.4989 vs 4.1296 m^2^/g), which is another factor that lowers its activity for PET glycolysis.

We investigated the possibility of recovery of the catalyst for its reuse in consecutive PET glycolysis reactions. To do this, we ran a reaction similar to the reaction L6 in Table 2 by using 2.0 wt % Cat-800 to account for the possible catalyst loss during the recovery process. The hot reaction mixture (as in Figure 2a) was vacuum-filtered on a preheated funnel, and the catalyst was recovered and dried in a vacuum oven at 65 °C overnight. Then, the recovered catalyst was used in a second PET glycolysis reaction. Our results showed that this catalyst loses its efficiency in the PET glycolysis reaction after being used in the first depolymerization reaction. The PET conversion reaches 84% in the second consecutive reaction using the recovered catalyst, most likely due to the inactivation of the active sites on the catalyst by the BHET or other components of the reaction.

### Analysis of the Reaction Mixture of PET Glycolysis

#### FTIR Analysis of the Crude Reaction Mixtures

**Figure 5:**
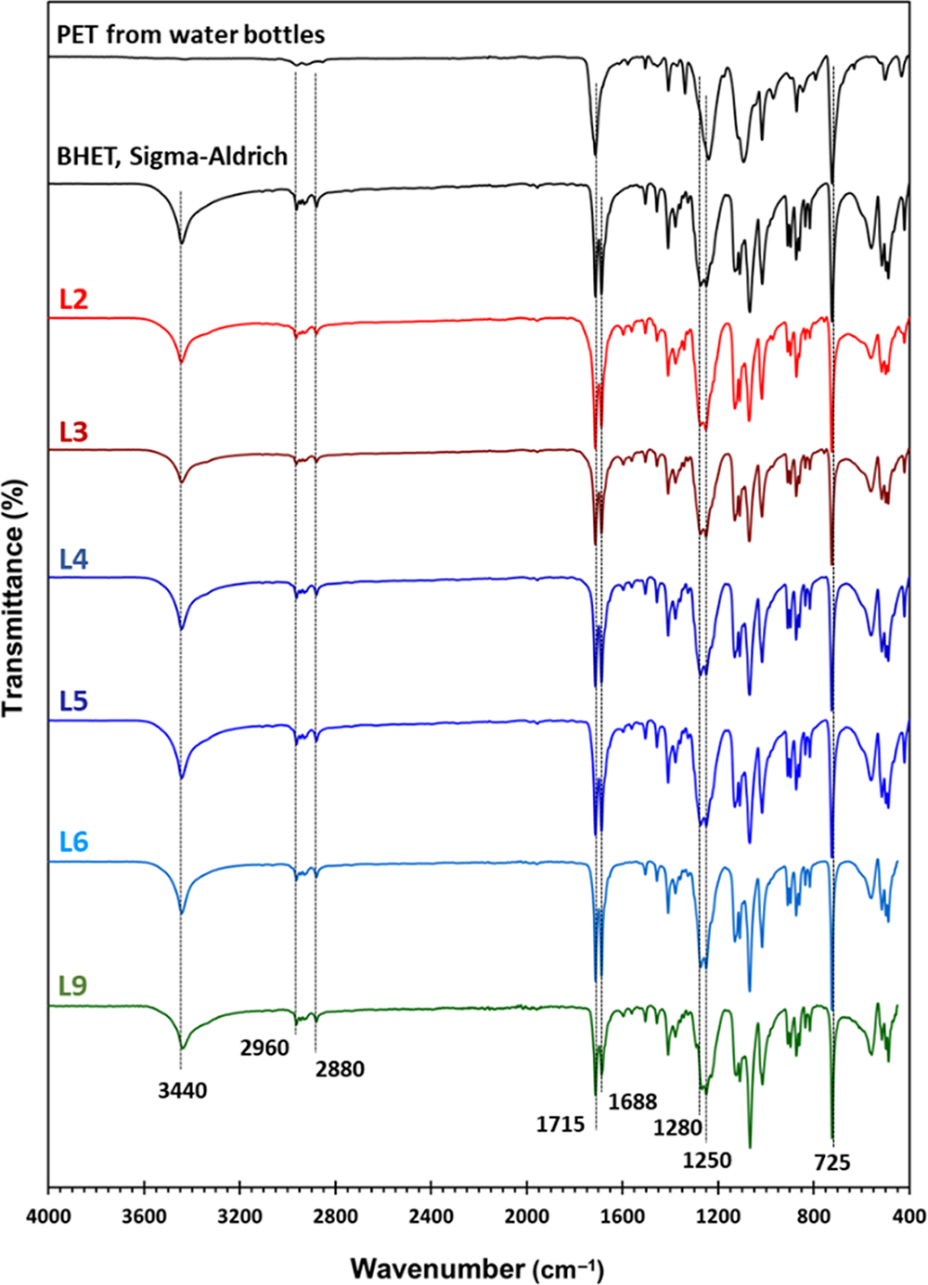
FTIR spectra of PET waste, pure BHET, and the crude reaction mixtures obtained from PET glycolysis (see Table 2 for reaction conditions).

FTIR spectroscopy is a fast and convenient way to qualitatively assess the reaction mixture by comparing the spectra of the crude reaction mixtures with the spectrum of pure BHET. Figure 5 shows the FTIR spectra of the PET waste, pure BHET, and some of the crude reaction mixture from PET glycolysis experiments using catalysts from waste labels. BHET shows characteristic FTIR peaks at 3440 cm^–1^ for the vibration of the hydroxyl (OH) group, 2960 and 2880 cm^–1^ for C–H stretching, 1715 cm^–1^ for carbonyl (C=O), 1688 cm^–1^ for aromatic C=C bending, 725 cm^–1^ for aromatic C–H bending, and 1250 and 1280 cm^–1^ for C–O–C bond vibration.^14,18^ As shown in Figure 5, all L2–L6 and L9 samples show the peaks associated with the BHET, as they contain 70.3–95.8% BHET in the crude reaction mixture (Table 2).

#### NMR Analysis of the Crude Reaction Mixtures

**Figure 6:**
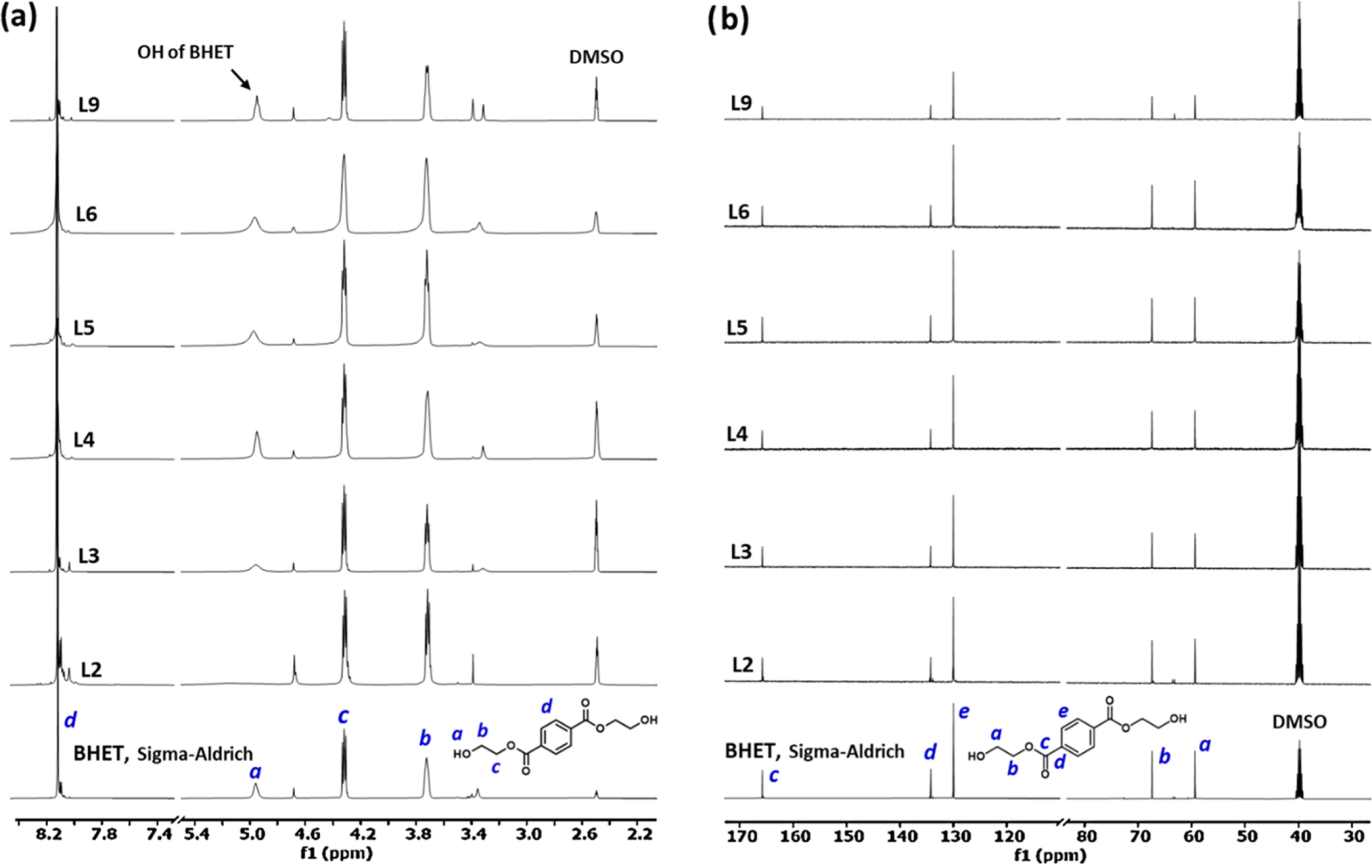
^1^H (a) and ^13^C NMR (b) spectra of pure BHET and some of the crude reaction products from PET glycolysis reactions (see Table 2 for the reaction conditions).

Figure 6 shows the ^1^H and ^13^C NMR spectra of pure BHET compared with those of samples from the reactions. Pure BHET shows peaks at 3.72 and 4.32 ppm for the methylene protons (b) and (c), 4.92 ppm for hydroxyl proton (a), and 8.12 ppm for aromatic protons (d) in its ^1^H NMR spectrum^41^ (Figure 6a). The BHET dimer and soluble higher oligomers show extra peaks for the internal ethylene glycol unit(s) at 4.69 ppm.^42−44^ The intensity and integral of this peak can be used as a measure of the efficiency of the catalyst and the glycolysis process because, in an ultimate glycolysis reaction, only BHET should be present. Sample L2 shows a moderately high intensity of the 4.69 ppm peak (Figure 6a). Since the PET conversion is 95% and the BHET yield is 70.3 for this sample, the remaining 24.7% of the converted PET must be oligomers. However, it should be mentioned that only BHET, dimer, and trimers of PET are soluble in DMSO-*d6*, and insoluble higher oligomers, therefore, cannot be detected in NMR using this solvent.^42^ Other samples with a higher BHET yield show lower peak intensities associated with dimer and higher oligomers, especially sample L6 with a 95.8% BHET yield that shows a ^1^H NMR spectrum of pure BHET (Figure 6a). The ^13^C NMR analysis of the samples (Figure 6b) is consistent with the ^1^H NMR results. Pure BHET shows peaks at 59.0, 67.0, 129.5, 133.8, and 165.2 ppm in its ^13^C NMR spectrum that is assigned to its carbon structure, as shown in Figure 6b.^43^ As for the ^1^H NMR, the presence of the BHET dimer and soluble higher oligomers resulted in some additional peaks in the ^13^C NMR spectrum, including at 63, 130, 134, and 165 ppm.^42,43^ The ^13^C NMR results for sample L2 with a higher oligomer show such additional peaks in addition to the BHET peaks, while samples with a high BHET yield, including L3–L6, show only the BHET peaks (Figure 6b).

#### DSC and TGA Characterization

**Figure 7:**
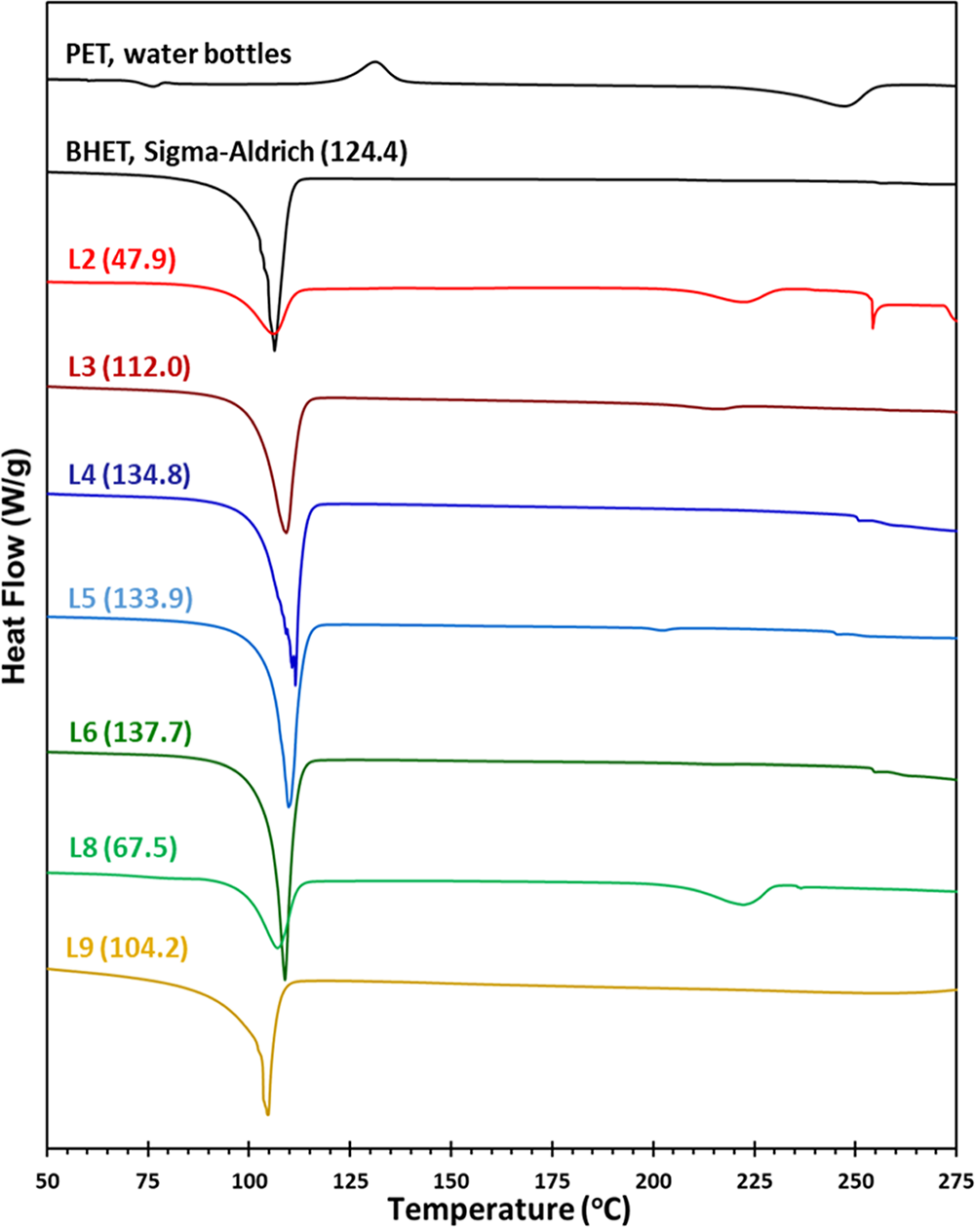
DSC thermograms of PET waste, pure BHET, and crude reaction mixtures obtained from PET glycolysis. Numbers in parentheses are the BHET melting enthalpies in J/g. (see Table 2 for reaction conditions).

Thermal analysis can be used to characterize the PET glycolysis reaction and to identify its product(s).^23,43,44^Figure 7 shows the DSC thermograms of the PET waste, pure BHET, and several crude reaction samples. PET shows its melting endotherm at 250 °C, and BHET has a melting endotherm at 107 °C, while BHET dimers and oligomers show melting peaks in between.^42−44^ Samples L2, L3, and L8 with lower BHET yields of 70.4, 70.5, and 64.6% show melting peaks at around 217 and 223 °C due to the presence of oligomers in addition to the BHET melting endotherm (unreacted PET was removed from the mixture before analysis). Samples L4, L5, L6, and L9 with high yields of the BHET show DSC thermograms very similar to the pure BHET. Sample L6, with a 95.8% BHET yield, shows a melting point of 110 °C and a sharper endotherm with a high enthalpy of melting (Figure 7).

**Figure 8:**
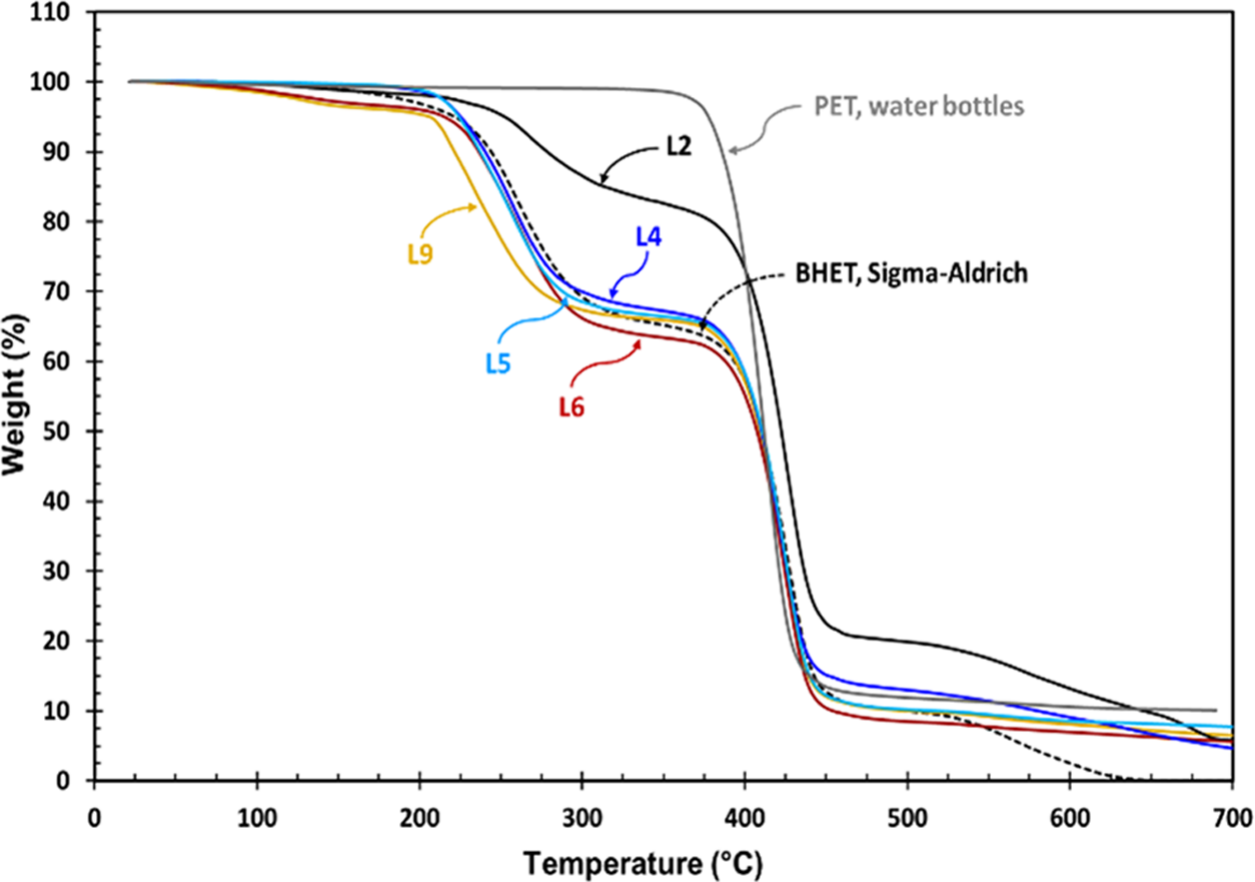
TGA thermograms of PET waste, pure BHET, and the crude reaction mixtures obtained from PET glycolysis (see Table 2 for reaction conditions).

Figure 8 shows the TGA thermograms of pure BHET compared to PET waste and some of the crude reaction mixtures. BHET shows three weight loss steps at temperature ranges of 210–270, 390–430, and 480–540 °C that correspond to about 34.0, 52.2, and 11.5% weight loss, respectively.^14^ The three weight losses of BHET can be attributed to the release of EG due to the oligomerization of BHET, decomposition of produced PET oligomers, and decomposition of the remaining BHET and possible produced PET. The TGA thermogram of the sample L2 with a 70.3% BHET yield shows characteristics of the BHET dimer,^43,45^ while samples L4, L5, L6, and L9 all show TGA thermograms very similar to the BHET, indicating a high amount of BHET in the mixture.

## Conclusions

By analysis of the chemical composition of solid additives used in packaging, we investigated the possibility of utilizing PET water bottle labels as a potential catalyst for the glycolytic depolymerization of PET. The solid additives used in the label manufacturing for PET water bottle packaging were used as a catalyst for the chemical recycling of the same PET waste. Three furnace temperatures of 600, 800, and 1000 °C were used for the catalyst preparation, which resulted in a solid catalyst recovery of 13.5, 12.0, and 10.4 wt %, respectively. The composition study and characterization of the catalyst showed the presence of CaCO_3_, CaO, and TiO_2_, which is expected because these are frequently used as fillers and/or pigments in the plastic industry. These catalysts were used in a typical glycolysis reaction using a high-pressure autoclave reactor, and the reaction mixture was analyzed for PET conversion and BHET monomer yield. While a 1.0 wt % loading of Cat-600 and Cat-1000 in the glycolysis reaction results in 71 and 93% PET conversion and 25.1 and 64.6% BHET yields, respectively, in 1 h, our results showed that Cat-800 is more efficient in PET glycolysis with a 1.0 wt % loading, as it leads to a PET conversion of 100% and a BHET yield of 81.9 and 95.8% in 1 and 1.5 h, respectively. While the catalyst can be isolated from the reaction mixture by filtration of the hot reaction mixture, the reuse of the recovered catalyst shows poor PET conversion. Additionally, calcium carbonate and titanium dioxide are both nontoxic and environmentally friendly materials, already widely used as additives for many plastic products; therefore, the 1.0 wt % catalyst obtained from the waste labels can be left in the reaction mixture without the need for further purification or reuse. In the case that pyrolysis of the labels is of interest, this catalyst can be considered as the byproduct of a waste management process for alleviating another waste issue, PET chemical recycling, thus creating a synergistic plastic recycling system.
